# Interplay of gene expression and regulators under salinity stress in gill of *Labeo rohita*

**DOI:** 10.1186/s12864-023-09426-x

**Published:** 2023-06-19

**Authors:** Vemula Harshini, Nitin Shukla, Ishan Raval, Sujit Kumar, Vivek Shrivastava, Aparna Chaudhari, Amrutlal K. Patel, Chaitanya G. Joshi

**Affiliations:** 1 Gujarat Biotechnology Research Centre, Sector 11, Gandhinagar, 382010 Gujarat India; 2 Postgraduate Institute of Fisheries Education and Research, Kamdhenu University, Himmatnagar, 383010 Gujarat India; 3 Central Institute of Fisheries Education, Mumbai, 400061 Maharashtra India

**Keywords:** *Labeo rohita*, Salinity stress, Gill transcriptome, ceRNA network

## Abstract

**Background:**

*Labeo rohita* is the most preferred freshwater carp species in India. The concern of increasing salinity concentration in freshwater bodies due to climate change may greatly impact the aquatic environment. Gills are one of the important osmoregulatory organs and have direct contact with external environment. Hence, the current study is conducted to understand the gill transcriptomic response of *L. rohita* under hypersalinity environment.

**Results:**

Comprehensive analysis of differentially expressed long non-coding RNAs (lncRNAs), microRNAs (miRNAs) and mRNAs was performed in gills of *L. rohita* treated with 2, 4, 6 and 8ppt salinity concentrations. Networks of lncRNA-miRNA-mRNA revealed involvement of 20, 33, 52 and 61 differentially expressed lncRNAs, 11, 13, 26 and 21 differentially expressed miRNAs in 2, 4, 6 and 8ppt groups between control and treatment respectively. These lncRNA-miRNA pairs were regulating 87, 214, 499 and 435 differentially expressed mRNAs (DE mRNAs) in 2, 4, 6 and 8ppt treatments respectively. Functional analysis of these genes showed enrichment in pathways related to ion transportation and osmolyte production to cope with induced osmotic pressure due to high salt concentration. Pathways related to signal transduction (MAPK, FOXO and phosphatidylinositol signaling), and environmental information processing were also upregulated under hypersalinity. Energy metabolism and innate immune response pathways also appear to be regulated. Protein turnover was high at 8ppt as evidenced by enrichment of the proteasome and aminoacyl tRNA synthesis pathways, along with other enriched KEGG terms such as apoptosis, cellular senescence and cell cycle.

**Conclusion:**

Altogether, the RNA-seq analysis provided valuable insights into competitive endogenous (lncRNA-miRNA-mRNA) regulatory network of *L. rohita* under salinity stress. *L. rohita* is adapting to the salinity stress by means of upregulating protein turnover, osmolyte production and removing the damaged cells using apoptotic pathway and regulating the cell growth and hence diverting the essential energy for coping with salinity stress.

**Supplementary Information:**

The online version contains supplementary material available at 10.1186/s12864-023-09426-x.

## Background

Salinity is one of the crucial environmental factors that affects fish survival. Hyper salinity of water can act as a stressor for freshwater fishes and directly influence the growth, development and reproduction [1, 2]. In recent years, climate change is leading to variations in temperature and precipitation patterns markedly [3] causing increased evaporation of freshwater bodies and eventually leading to increased salinization. Apart from this, excessive use of groundwater, rise in sea water level, pollution from anthropogenic activities, frequent flooding and loss of annual rainfall are few other reasons contributing towards rise of water salinity level [4].

In India, carp culture is the backbone of freshwater aquaculture and it is compatible with other farming systems. Among carps, *Labeo rohita* (rohu), is well distributed in India and South Asia, and found to significantly contribute to total production when cultured with other Indian major carp species [5]. Rohu has significantly higher muscle protein content than other carp species and consumer demand, which made it economically important species [6]. Fishes are dependent on an effective osmoregulatory mechanism for body fluid homeostasis [7] and among osmoregulatory organs, gills play an important role because of their large surface area and direct contact with external aquatic environments [8, 9]. Studies have identified that gill filaments of fishes have mitochondria rich cells (MRCs), which can increase the ion regulation capacity of gills in response to altered osmotic challenges [10, 11].

Regulatory non-coding RNAs are classified into short non-coding RNAs (microRNAs) and long non-coding RNAs (lncRNAs) based on transcript length [12]. MicroRNAs (miRNAs) are 22 nucleotide (nt) length transcripts and regulates mRNA expression at post-transcriptional level [13], whereas lncRNAs are more than 200 nt in length and regulates mRNAs expression at transcriptional and post-transcriptional level [14]. miRNA response elements of lncRNAs interact with miRNA and indirectly regulate mRNAs. The concept of competitive endogenous (ceRNA) hypothesis was proposed by Salmena and co-workers, which demonstrates that lncRNAs can act as endogenous sponge to regulate mRNA expression by sinking miRNA [15, 16]. There were studies focused on the role of miRNAs in osmotic pressure regulation [17], salinity stress [18] and immune response [19] and lncRNAs under adverse environmental stress conditions [20]. A recent study on integrated analysis of lncRNA-miRNA-mRNA in Atlantic salmon was conducted to identify the potential regulators of immune response challenged by pathogenic bacteria *Aeromonas salmonicida* [19]. However, the integrated role of lncRNA-miRNA-mRNAs of *Labeo rohita* under salinity stress remains unexplored.

In this concern, we constructed 16 gill transcriptome libraries and integrated analysis of lncRNAs, miRNAs and mRNAs in *Labeo rohita* treated with various salinity concentrations in comparison with the control group. Eventually, the ceRNA network was constructed and performed functional enrichment analysis of differentially expressed mRNAs involved in the network. In addition, this comprehensive analysis also provides hints to find genes with active role in stress response of *L. rohita* under hyper salinity conditions.

## Result

### Gill transcriptome profile of *Labeo rohita*

A total of 335 Gbp raw data was generated from gill transcriptome of control and salinity treated fingerlings. After filtering low quality sequences, 57,574,643 and 47,647,565 million clean reads are obtained in control and treatment groups, respectively. The raw transcriptome sequence data is submitted to NCBI Short Read Archive (SRA) (supplementary table S1). Mapping of the clean reads with reference genome revealed mapping percentages varied from 77.56 to 90.9% (supplementary table S1). Sample-wise summary statistics of transcriptome sequences are represented in supplementary table S1.

### Differential expression of mRNA under salinity stress

**Fig. 1:**
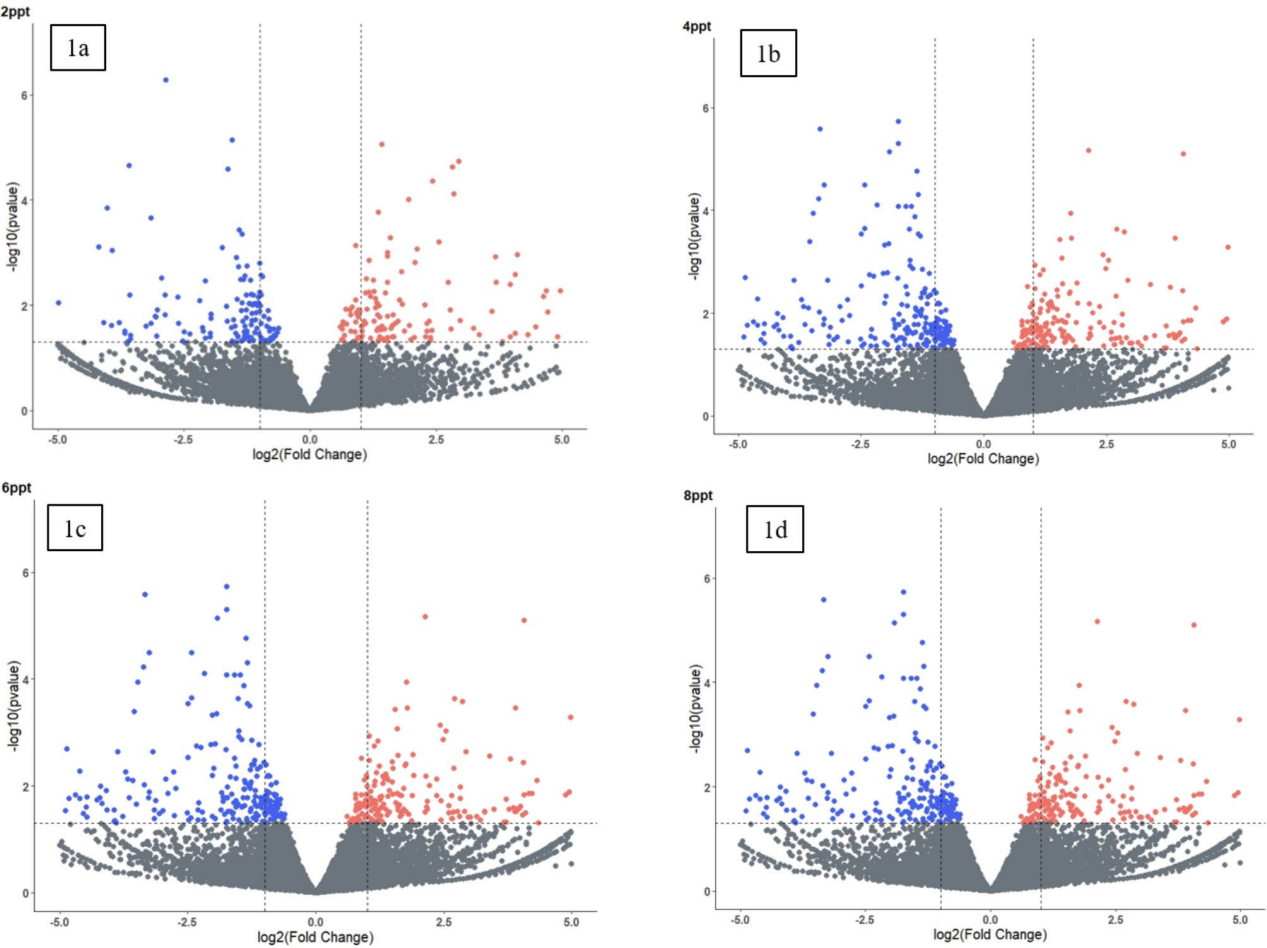
Volcano plot of differentially expressed genes identified between control and 2ppt (**1a**), 4ppt (**1b**), 6ppt (**1c**) and 8ppt (**1d**) salinity treated *L. rohita.* The X-axis signifies Log2FoldChange value and Y-axis signifies –Log10 p-value. The ash color dots indicates non-significant genes, blue dots indicates significantly down regulated and orange dots indicates significantly up regulated genes

In the gill of *L. rohita* under salinity stress, 363, 532, 836 and 892 transcripts showed differential expression at 2, 4, 6 and 8ppt salinity concentrations respectively in comparison with the control. A complete list of genes differentially expressed along with Log2FC, and p-values are given in supplementary table S2 for 2, 4, 6 and 8ppt salinity treated groups. The volcano plots visualizing the differentially expressed genes with statistically significant fold change are given in Fig. 1a, b, c and d for 2, 4, 6 and 8ppt treatments respectively.

### Identification and pathway enrichment of hub genes

**Fig. 2:**
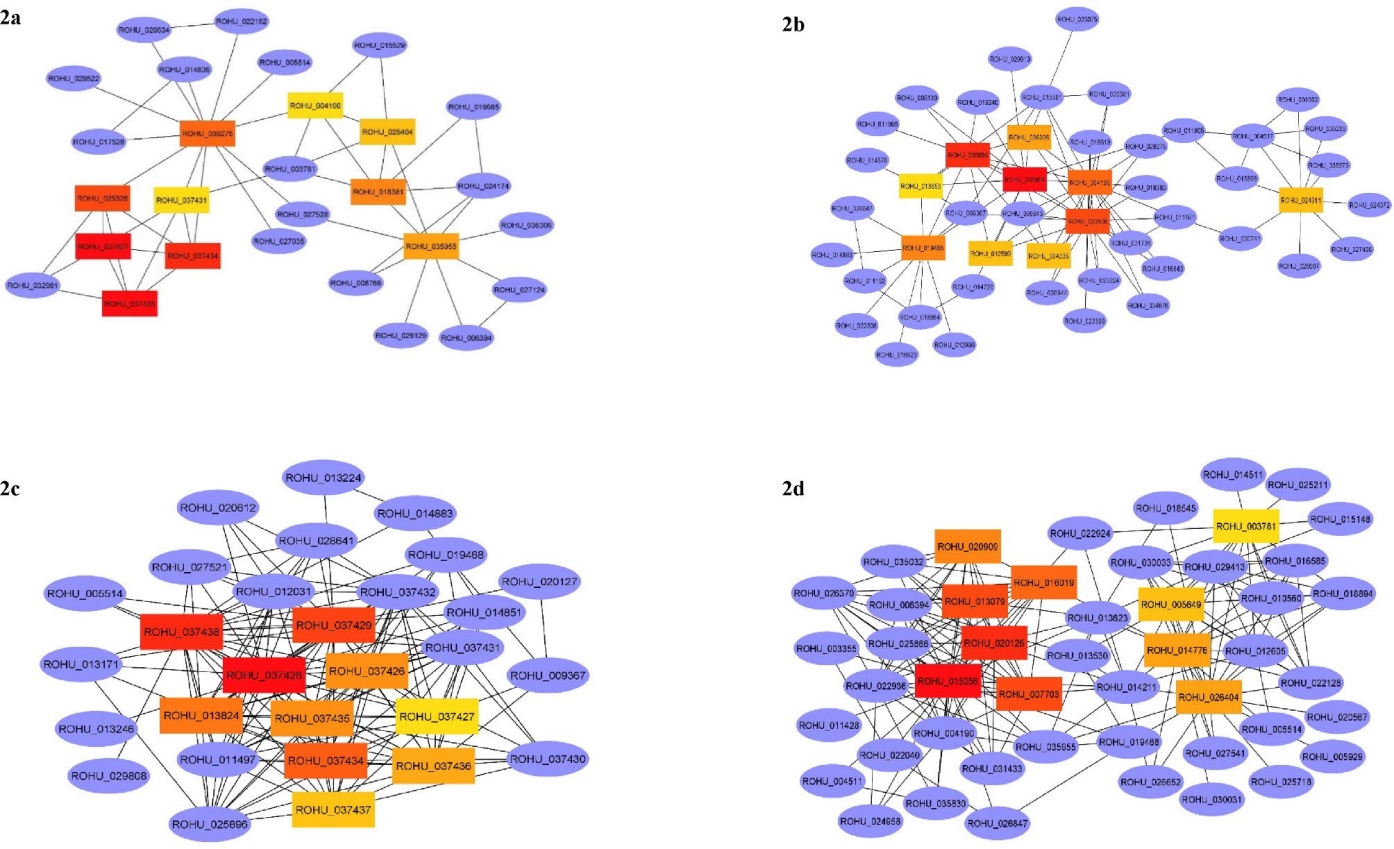
Hub genes identified using CytoHubba plugin with maximal clique centrality (MCC) algorithm on Cytoscape for 2ppt (**2a**), 4ppt (**2b**), 6ppt (**2c**) and 8ppt (**2d**) salinity treatments. Edges represent protein-protein interaction. Red nodes represent genes with highest MCC score and yellow nodes represent genes with low MCC scores. Blue nodes represent the genes that are directly interacted with hubgenes

Top 10 hub genes identified from PPI network for 2, 4, 6 and 8ppt salinity concentrations are presented in supplementary table S3 (Fig. 2). KEGG pathway enrichment of hub genes is given in Table 1. Most of the hub genes in 2, 4 and 6ppt salinity treatments found to be involved in aminoacyl-tRNA biosynthesis, ATP production, metabolic pathways and osmolyte production, while in 8ppt treatment group hub genes involved solely in proteasome and aminoacyl-tRNA biosynthesis.

**Table 1:** KEGG pathway enrichment of top 10 hub genes identified in 2, 4, 6 and 8ppt gill transcriptome DEGs network analysis of *Labeo rohita* under hyper salinity stress

2ppt (p-value)	4ppt (p-value)	6ppt (p-value)	8ppt (p-value)
Oxidative phosphorylation (**5.01E-08**) Aminoacyl-tRNA biosynthesis (**2.87E-07**) Metabolic pathways (**4.03E-05**) Selenocompound metabolism (**0.0047**) Progesterone-mediated oocyte maturation (**0.038**)	Progesterone-mediated oocyte maturation (**0.0008**) ErbB signaling pathway (**0.0009**) Necroptosis (**0.001**) NOD-like receptor signaling pathway (**0.001**) Protein processing in endoplasmic reticulum (**0.002**) Fructose and mannose metabolism (**0.014**) Glycolysis / Gluconeogenesis (**0.02**) Biosynthesis of amino acids (**0.02**) Carbon metabolism (**0.03**) Inositol phosphate metabolism (**0.03**) ECM-receptor interaction (**0.04**) GnRH signaling pathway (**0.04**)	Oxidative phosphorylation (**3.68E-24**) Metabolic pathways (**1.47E-13**) Cardiac muscle contraction (**8.83E-08**)	Proteasome (**1.71E-14**) Aminoacyl-tRNA biosynthesis (**1.11E-09**)

### Identification of differentially expressed miRNAs and their target genes

BLASTn against mature miRNA sequences of teleostei species available on miRBase (Release 22.1) revealed several potential known miRNA hits against control and salinity treated gill transcriptome sequences (supplementary table S4). Differential expression of several miRNAs is observed in salinity treatments compared to controls (Table 2). A total of 284 i.e. 154 down and 130 up regulated, 444 i.e. 239 down and 205 up regulated, 681 i.e. 217 down and 464 up regulated and 738 i.e. 308 down and 430 up regulated DE mRNAs are targeted by DE miRNAs in 2, 4, 6 and 8ppt salinity treatments respectively (supplementary table S5). Several up and down regulated mRNAs are commonly targeted by different DE miRNAs are observed in four treatments.

**Table 2:** Differentially expressed miRNAs in gill transcriptome of *Labeo rohita* in 2, 4, 6 and 8ppt treatments along with log2fold change and p-values

2ppt			4ppt			6ppt			8ppt		
**miRNA**	**Log2** **FC**	**p-value**	**miRNA**	**Log2** **FC**	**p-value**	**miRNA**	**Log2** **FC**	**p-value**	**miRNA**	**Log2** **FC**	**p-value**
tni-let-7i oni-miR-10,805 hhi-miR-7793 oni-miR-10,798 dre-miR-152 ssa-miR-139-5p dre-miR-725-3p ipu-miR-7562 dre-miR-725-5p tni-miR-17 pol-miR-203-5p ssa-miR-7132a-3p ssa-miR-193-3p ssa-miR-142a-3p	-6.74 3.40 -2.47 -6.32 -5.94 -5.84 -5.65 -5.63 -5.63 1.65 -5.43 -5.45 3.38 -2.44	0.000 0.000 0.001 0.002 0.008 0.011 0.022 0.025 0.025 0.029 0.040 0.044 0.047 0.048	dre-miR-732 oni-miR-10547c dre-miR-152 oni-miR-10,690 dre-miR-451 oni-miR-10,926 oni-miR-10,902 dre-miR-430c-3p gmo-miR-11937-3p ssa-miR-27a-5p ssa-miR-142a-3p ssa-miR-20b-3p oni-miR-10,815 oni-miR-449b-5p oni-miR-10,966 dre-miR-181b-3-3p oni-miR-10,582 dre-miR-7132-5p	-24.08 -5.76 -3.94 -7.67 -2.51 -4.71 -2.25 -2.53 -1.36 4.10 2.90 3.35 -4.08 5.28 -1.14 1.00 -2.74 -2.14	0.000 0.000 0.000 0.000 0.001 0.002 0.013 0.014 0.020 0.021 0.025 0.029 0.030 0.034 0.034 0.039 0.043 0.044	dre-miR-732 tni-miR-216b ssa-miR-128-3-5p ipu-miR-205 tni-miR-128 ssa-miR-462a-5p nbr-miR-7133-3p gmo-miR-459-3p ssa-miR-93a-3p eel-miR-7132-3p gmo-miR-187-5p dre-miR-128-3-5p tni-miR-22a oni-miR-10,796 oni-miR-10,815 ssa-miR-146a-3p ipu-miR-19a dre-miR-152 oni-miR-10,744 oni-miR-10,826 ssa-miR-301b-5p ssa-miR-200a-1-5p oni-miR-728b ssa-miR-7a-2-3p abu-miR-10556b oni-miR-10,671 oni-miR-10,698 oni-miR-10,632 oni-miR-10,580	-23.88 7.85 -7.21 -6.86 6.90 -6.29 -6.36 -6.21 -6.21 -6.13 3.72 -6.13 -6.10 6.28 -6.43 -5.95 -5.83 -5.83 6.45 3.44 -5.73 -5.76 -5.70 -3.82 -5.67 -5.64 -5.71 -5.60 -5.57	0.000 0.000 0.001 0.003 0.004 0.011 0.012 0.013 0.013 0.016 0.017 0.017 0.018 0.020 0.023 0.023 0.029 0.029 0.032 0.032 0.035 0.035 0.037 0.038 0.040 0.041 0.042 0.044 0.047	gmo-miR-11930-3p ipu-miR-27d pny-miR-135c-3p ssa-miR-462a-5p tni-miR-21 tni-miR-9 oni-miR-10,712 oni-miR-10,737 gmo-miR-33b-2-3p oni-miR-10,873 ssa-miR-106a-3p oni-miR-10,778 abu-miR-33-5p ssa-miR-19c-5p ipu-let-7j gmo-miR-11262-3p gmo-miR-11224b-3p ssa-miR-27a-3p dre-miR-24b-5p dre-miR-732 dre-miR-725-3p	-11.35 -7.19 -6.99 -6.73 -6.59 -4.13 -4.88 -6.42 -6.41 -6.30 -6.20 -6.08 -6.02 -6.02 4.83 -5.94 -5.94 -5.95 -5.89 -9.43 2.69	0.000 0.001 0.004 0.006 0.011 0.015 0.015 0.015 0.018 0.019 0.026 0.031 0.035 0.035 0.037 0.042 0.042 0.045 0.046 0.048 0.049

### miRNA-mRNA regulatory network

miRNA-mRNA regulatory network is constructed based on the differential expression of miRNA-mRNA pairs obtained (supplementary table S5) for 2, 4, 6 and 8ppt salinity treatments against the controls. There are 1490 (14 miRNAs and 284 DE mRNAs), 1470 (18 miRNAs and 444 mRNAs), 2639 (29 miRNAs and 681 mRNAs) and 4988 (21 miRNAs and 738 mRNAs) miRNA-mRNA pairs identified in 2, 4, 6 and 8ppt treatments respectively. For clear visualization of the network, miRNAs with p-value < 0.01 and their target DE mRNAs are considered for construction of the network (supplementary figures SF1 and SF2).

### Identification and characterization of lncRNA

**Fig. 3:**
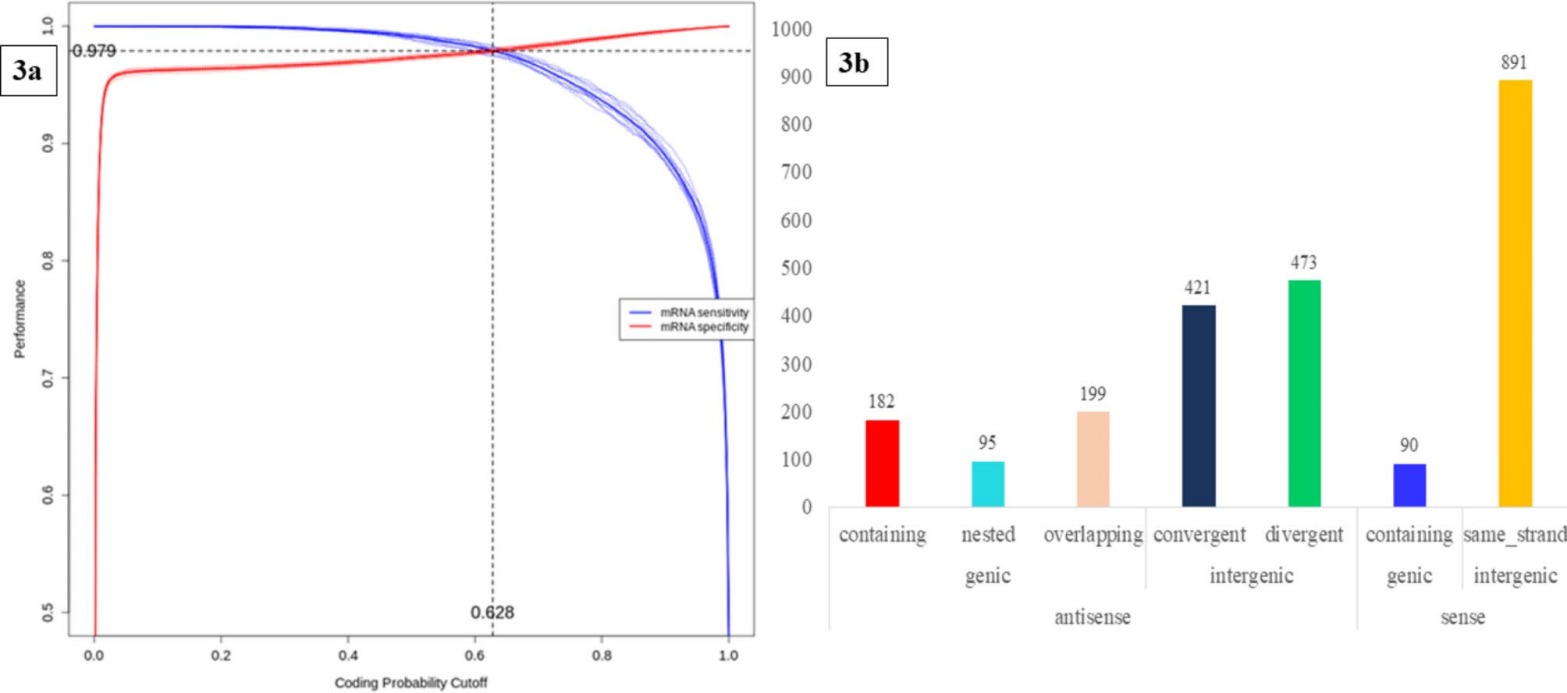
**a**) Two-graph ROC curve where red line indicate specificity of mRNA and blue line indicates specificity of lncRNA **b**) Different classes of lncRNA identified in gill transcriptome of *Labeo Rohita*

More than 86 million mapped reads were utilized to merge > 37,000 transcripts using Cuffmerge. A total of 8,710 putative transcripts with non-coding potential are identified through FEELnc_codpot_ with 0.979 specificity and sensitivity and further, error of 0.021 is reduced by filtering the data through FEELnc_filter_ (Fig. 3a). The identified transcripts are further filtered through CPC2 (v0.1), retaining 2,490 with probable potential non-coding transcripts. The transcripts are classified through FEELnc_classfier_ resulting in 566 genic and 1785 intergenic lncRNAs using cutoff score of 1 (Fig. 3b). The generated GTF, BED and FASTA files are used for differential expression of lncRNAs in 2, 4, 6 and 8ppt treatments. A total of 55, 76, 88 and 136 differentially expressed lncRNAs with p-value < 0.05 | log2 FoldChange > 0.5 are obtained in 2, 4, 6 and 8ppt treatments respectively (supplementary table S6).

### Prediction of lncRNA-miRNA pairs

A total of 109 lncRNA-miRNA pairs consisting of 14 miRNAs and 40 lncRNAs are obtained in 2ppt treatment, while in 4ppt treatment 87 lncRNA-miRNA pairs consisting of 16 miRNAs and 54 lncRNAs. Similarly, in 6ppt treatment, there are 181 lncRNA-miRNA pairs including 29 miRNAs and 69 lncRNAs, whereas in 8ppt treatment there are 195 lncRNA-miRNA pairs with 21 miRNAs and 94 lncRNAs (supplementary table S7).

### Identification of lncRNA-mRNA pairs and construction of lncRNA-miRNA-mRNA network

**Fig. 4:**
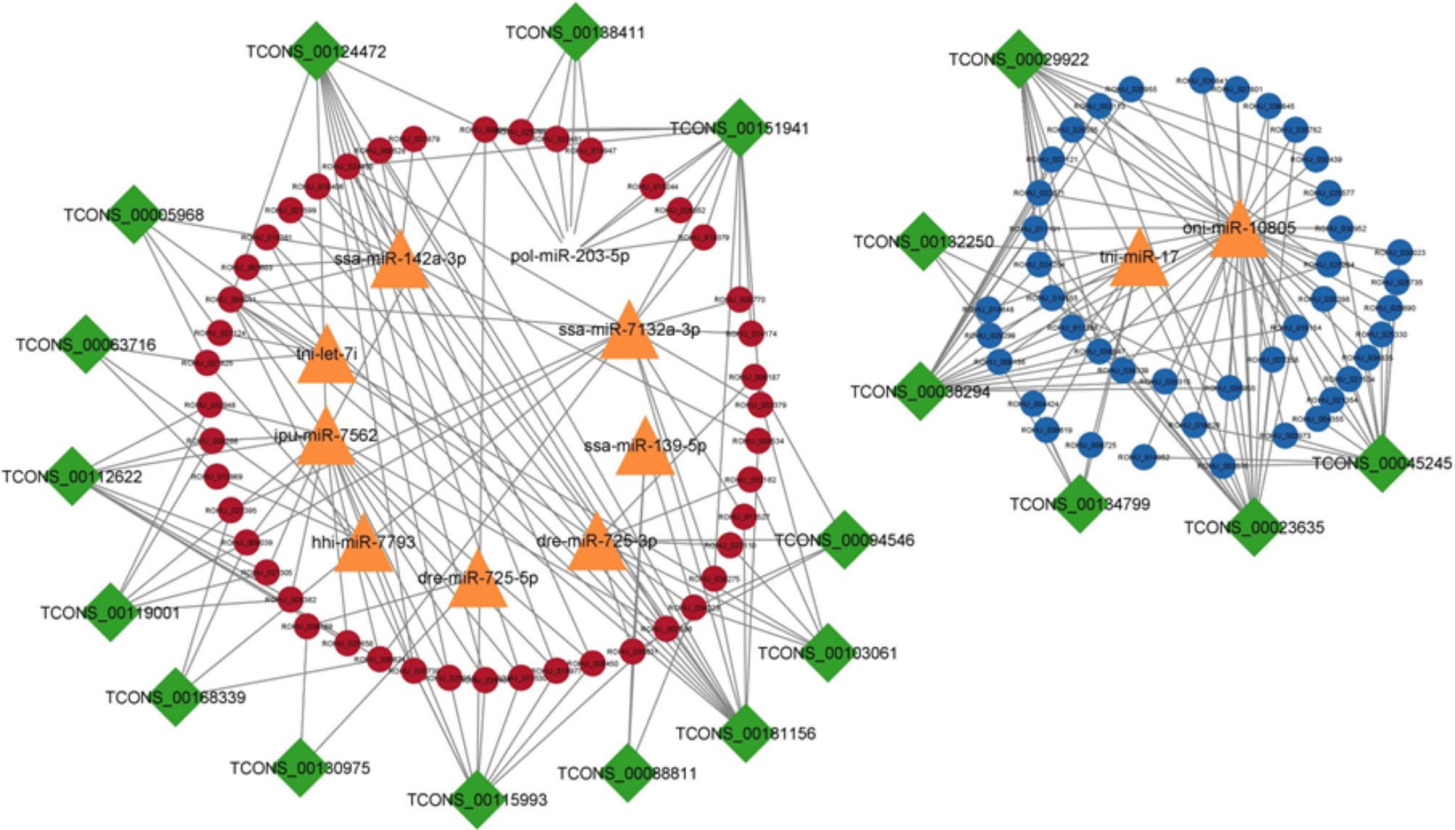
lncRNA-miRNA-mRNA integrated network of 2ppt treatment. Green colour diamonds, orange colour triangles, and circles represent lncRNA, miRNA and mRNA respectively. Red and blue colour circles represent up and down regulated mRNAs respectively. Network includes 259 edges and 118 nodes with 20, 11and 87 lncRNAs, miRNAs and mRNAs respectively

**Fig. 5:**
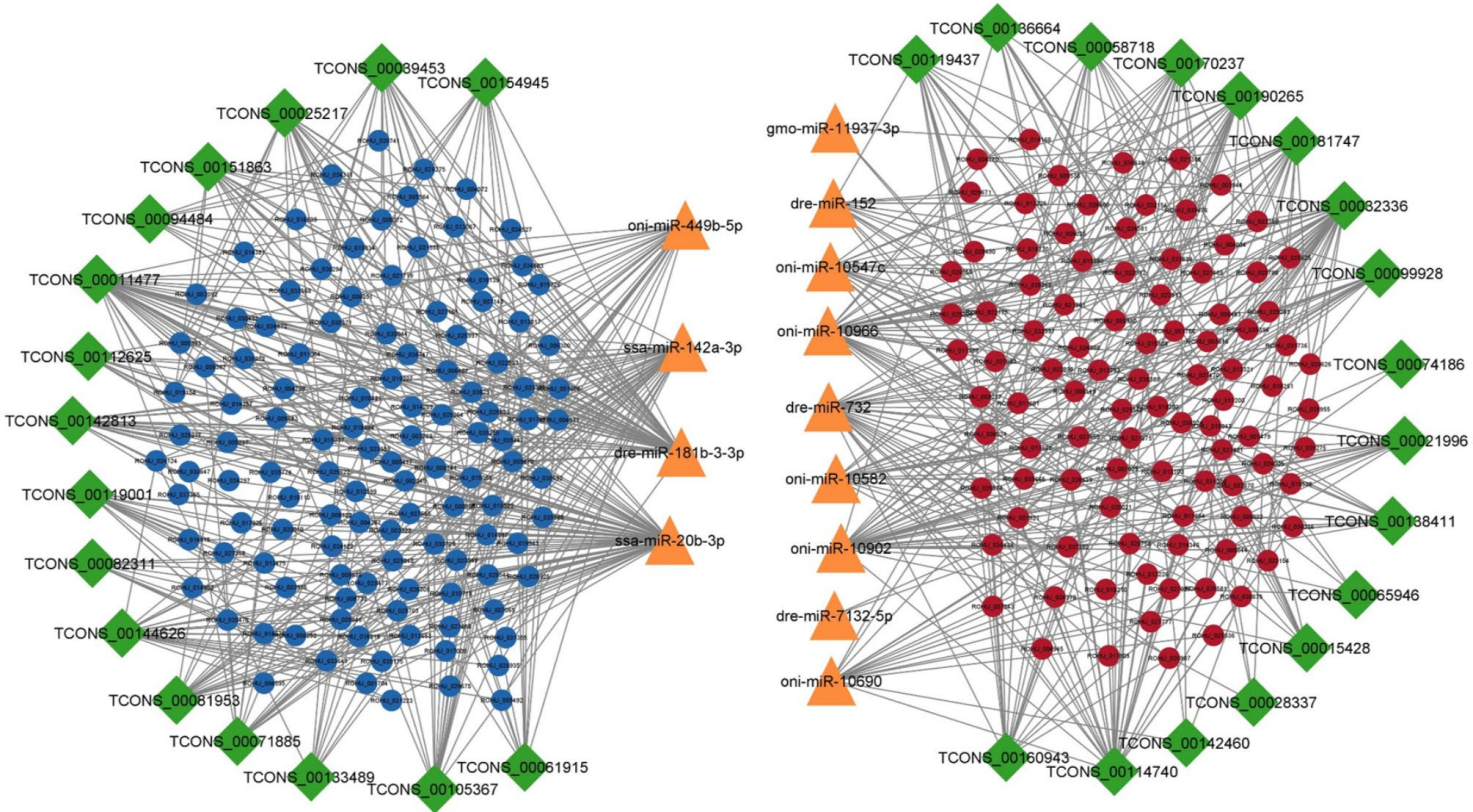
lncRNA-miRNA-mRNA integrated network of 4ppt treatment. Green colour diamonds, orange colour triangles, and circles represent lncRNA, miRNA and mRNA respectively. Red and blue colour circles represent up and down regulated mRNAs respectively. Network includes 824 edges and 260 nodes with 33, 13 and 214 lncRNAs, miRNAs and mRNAs respectively

**Fig. 6:**
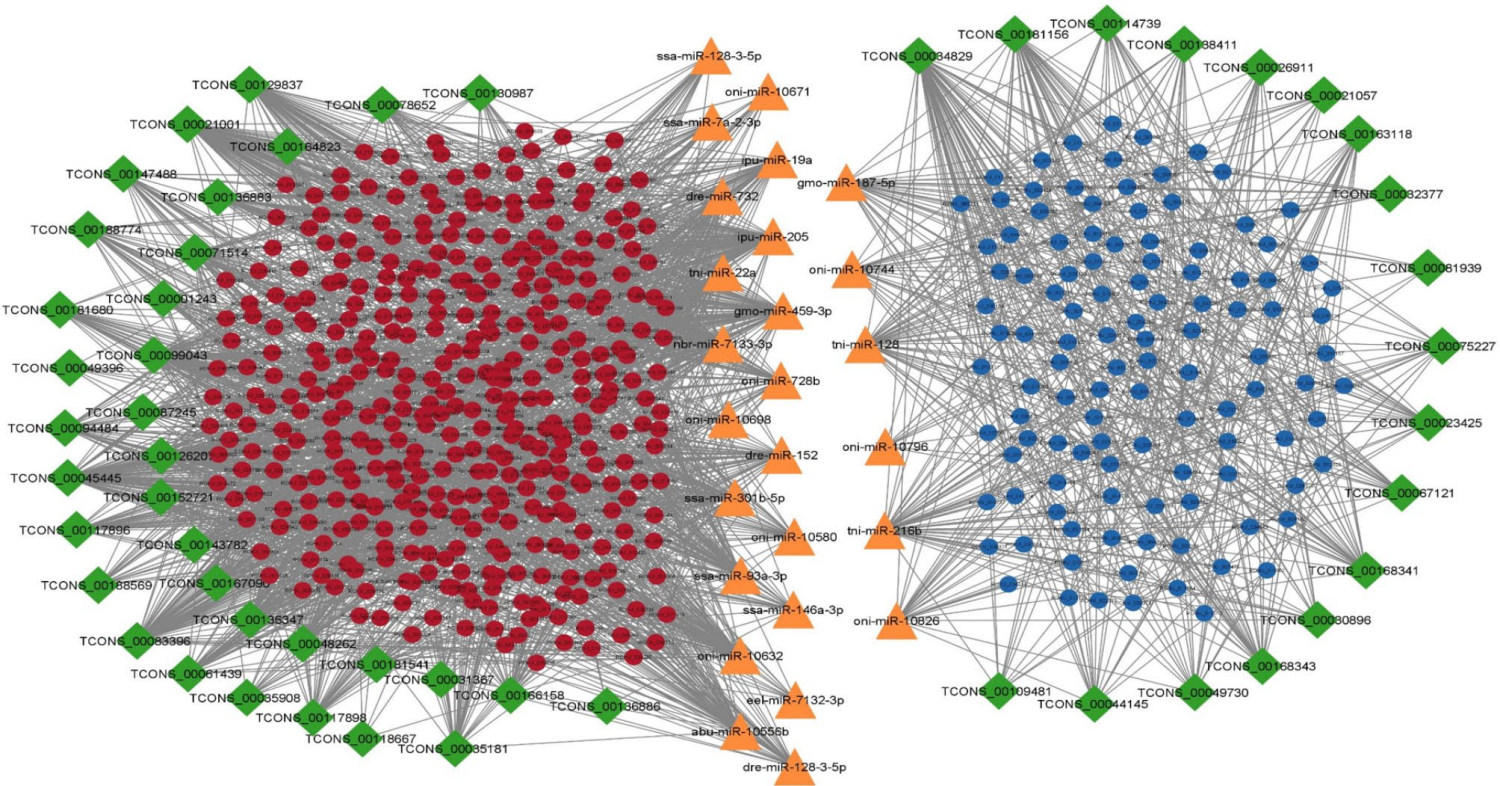
lncRNA-miRNA-mRNA integrated network of 6ppt treatment. Green colour diamonds, orange colour triangles, and circles represent lncRNA, miRNA and mRNA respectively. Red and blue colour circles represent up and down regulated mRNAs respectively. Network includes 2688 edges and 577 nodes with 52, 26 and 499 lncRNAs, miRNAs and mRNAs respectively

**Fig. 7:**
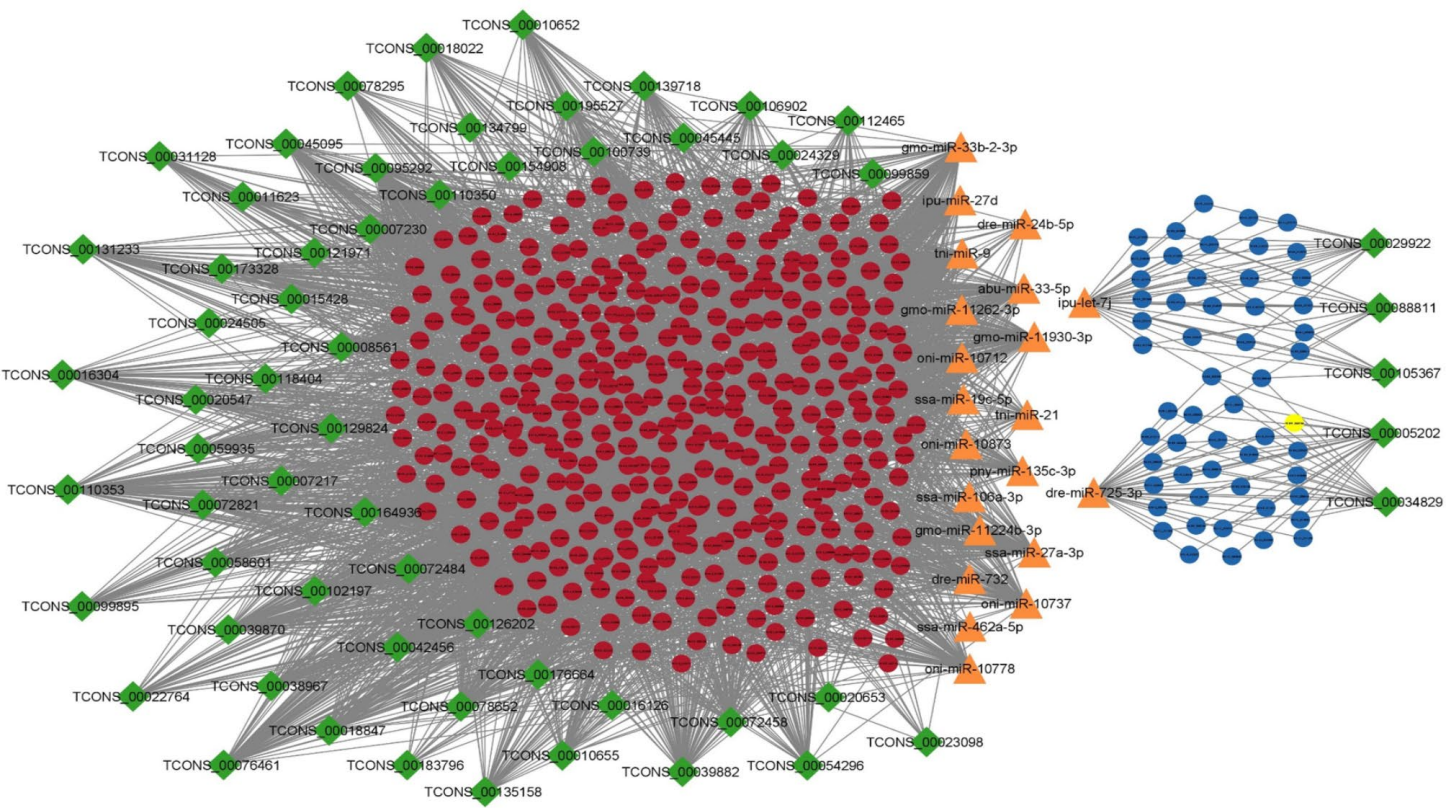
lncRNA-miRNA-mRNA integrated network of 8ppt treatment. Green colour diamonds, orange colour triangles, and circles represent lncRNA, miRNA and mRNA respectively. Red and blue colour circles represent up and down regulated mRNAs respectively. Network includes 3533 edges and 517 nodes with 61, 21 and 435 lncRNAs, miRNAs and mRNAs respectively

There were 2232, 4666, 12,549 and 18,926 lncRNA-mRNA pairs obtained with PCC < 0.90 and p value > 0.05 in 2, 4, 6 and 8ppt treatments respectively (supplementary table S8). In 2ppt treatment, 55 lncRNAs and 353 mRNAs, 4ppt treatment, 76 lncRNAs and 522 mRNAs, 6ppt treatment 88 lncRNAs and 835 mRNAs and in 8ppt treatment 136 lncRNAs and 889 mRNAs involved in lncRNA-mRNA pairs. According to the ceRNA hypothesis, ceRNAs (lncRNA and mRNA) have positive correlation expression by competing for the same miRNA, which is negatively co-expressed. Thus, two different lncRNA-miRNA-mRNAs pairs i.e. (1) upregulated lncRNAs and mRNAs, which were targeted by common down regulated miRNAs and (2) down regulated lncRNAs and mRNAs, which were targeted by common up regulated miRNAs, were identified and constructed networks (Figs. 4, 5, 6 and 7).

A total of 140 lncRNA-miRNA-mRNA pairs including 20 lncRNAs, 11 miRNAs and 87 mRNAs, 513 lncRNA-miRNA-mRNA pairs including 33 lncRNAs, 13 miRNAs and 214 mRNAs, 1860 lncRNA-miRNA-mRNA pairs including 52 lncRNAs, 26 miRNAs and 499 mRNAs and 2701 lncRNA-miRNA-mRNA pairs including 61 lncRNAs, 21 miRNAs and 435 mRNAs were selected in 2, 4, 6 and 8ppt treatments respectively (supplementary table S9).

### Functional enrichment of DE mRNAs involved in lncRNA-miRNA-mRNA network

The complete list of lncRNAs, miRNAs and mRNAs enriched in various KEGG terms are given in supplementary table S10. There was enrichment of KEGG terms involved in osmoregulation in *L. rohita* under hypersalinity stress. Differentially expressed mRNAs i.e. *SLC2A1*, *SLC9A3*, *SLC9A5*, *SLC15A5*, *SLC40A1*, *SLC12A9*, *SLC24A3*, *SLC24A4* and *ATP1A1* were found to be involved in KEGG terms related to ion transportation. We observed that TCONS_0071514 and ipu-miR-205 miRNA pair regulated *SLC2A1* and *ATP1A1* genes, whereas TCONS_00034829 and tni-miR-216b lncRNA-miRNA pair interacted with *SLC9A3* and *SLC40A1*. ssa-miR-142a-3p miRNA found to be negatively co-expressed with *SLC9A5* and *SLC12A9* mRNAs. The genes such as *ISYNA1*, *SLC5A3* and *SLC24A3* were regulated by oni-miR-10712. Genes involved in arachidonic pathway were commonly regulated by tni-miR-9 and abu-miR-33-5p miRNAs, whereas expression of aquaporin-8, gap junction like protein, *PDIA4* and *CAPN1*were potentially targeted by dre-miR-732 miRNA.

*UBE2D4*, *MAN1B1* and *ERO1* alpha genes involved in unfolded protein response were negatively co-expressed with ssa-miR-7a-2-3p, gmo-miR-187-5p and gmo-miR-459-3p respectively. TCONS_00007230-tni-miR-9 pair observed to be interacted with heat shock protein 90 alpha and *SEC23B*, while TCONS_00071514-ipu-miR-205 pair interacted with heat shock 70 kDa, *NFE2L2* and *CKAP4* mRNAs. There was also enrichment of DE mRNAs in cellular senescence, cell cycle and apoptosis pathways. Among these pathways, TCONS_00100739- gmo-miR-11930-3p pair regulated expression of *HLA-A* and *CDKN1B*, TCONS_00008561 lncRNA through different miRNAs regulated the *GADD45B* and *PPP3CA* and oni-miR-10632 interacted with *E2F1*, *MR1* and *RAD1*. gmo-miR-459-3p also interacted with *SMAD* like protein. The key gene in apoptosis pathway, *BAX* like protein, was regulated by TCONS_00167090- ssa-miR-93a-3p lncRNA-miRNA pair.

dre-miR-152 mediated the expression of *ELOVL6*, *FFAR3*, *ENO1* and *GYG1* genes of energy metabolism, whereas nbr-miR-7133-3p mediated expression of fatty acid synthase and phosphomannomutase 1. TCONS_00139718 and gmo-miR-33b-2-3p lncRNA-miRNA pair interacted with *IDH2* and *ACSL4* genes. *PDHA1* was solely regulated by gmo-miR-11930-3p, while *ACSS2* gene was regulated by pny-miR-135c-3p and gmo-miR-11224b-3p.

Interestingly, we observed that tni-miR-9 miRNA differentially co-expressed with mRNAs i.e. *C3AR1*, *C3*, *GRM6*, *GRM5*, *GRIN2A*, *TRPM2*, *IL1RAP*, *TNFSF14*, *COL1A1* and *PIGR*, involved in pathways such as ECM-receptor interaction, cell adhesion molecules, neuroactive ligand-receptor interaction and cytokine-cytokine receptor interaction. gmo-miR-459-39 mediated the expression of *RXFP1*, *TRPC1*, *S1PR1* and *CDH2* genes. TCONS_00024329-oni-miR-10712 pair regulated the two of the key genes, *BCL2* and *LEP*, involved in environmental information processing pathways. There was enrichment of DE mRNAs involved in various signaling pathways such as MAPK signaling, FOXO signaling, phosphatidylinositol signaling system and p53 signaling. Crucial mRNAs, *CASP3*, *CCNGC2*, *GGT1*, *HSP70*, *TBX2*, *SLC2A1* and *IGFBP1*, involved in MAPK, p53 and FOXO signaling pathways found to be regulated by specific TCONS_00071514- ipu-miR-205 lncRNA-miRNA pair, whereas *PRKCA* and *FGFR1* regulated by TCONS_00058718- oni-miR-10966 pair. gmo-miR-459-39 miRNA also regulated the *CTSL* and *AZGP1* genes involved in the phagosome pathway. tni-miR-9 also regulated the genes (*NLRP3*, *GBP1* and *TRPM2*) involved in the immune response pathway NOD-like receptor signaling pathway.

## Discussion

*Labeo rohita* is a freshwater fish, which maintains the body fluid homeostasis by absorbing salts and excreting water [21]. In the present study, *L. rohita* was reared in a hyper saline environment with higher salinity concentrations including 2, 4, 6 and 8ppt. As there was change in environmental salinity, fish made a regulatory shift from absorption to secretion [22]. This change was supported by the differential expression of several solute carrier family genes including *SLC2A1*, *SLC9A3*, *SLC9A5*, *SLC15A5*, *SLC40A1*, *SLC12A9*, *SLC24A3*, *SLC24A4* and *ATP1A1*. Inositol-3-phosphate synthase 1 A (*ISYNA1*) is rate limiting enzyme in production of myo-inositol [23]. Up regulation of the *ISYNA1* gene in our study reflected the osmolyte production in *L. rohita* to counterbalance the osmotic pressure induced due to hyper salinity stress. In addition, there was upregulation of sodium-myo inositol cotransporter-like protein (*SLC5A3*). Apart from this, there was also enrichment of differentially expressed genes in arachidonic acid metabolism. Arachidonic acid and its metabolites were reported to be involved in osmoregulatory process and response to confinement stress [24].

The changed solute concentration of body fluids caused proteins to lose their three dimensional structure and unfold, which led to unfolded protein response [25, 26] in rohu. The differential expression of several isoforms of heat shock proteins (HSP) 70 genes reflected the prevention of misfolded proteins from aggregation [27], while up regulated *UBE2D4*, *MAN1B1*, *ERO1* alpha, heat shock protein 90 alpha, *SEC23B*, *NFE2L2*, *NEDD4* and *CKAP4* mRNAs enriched in protein processing in endoplasmic reticulum and endocytosis pathways explained the degradation of ubiquitinated proteins through an ATP-dependent mechanism [28]. Up regulated mannosyl-oligosaccharide alpha-1,2-mannosidase (*MAN1A1*) gene reported to be accelerated ER associated degradation of misfolded proteins [29, 30]. Degradation of misfolded or unfolded proteins might help the individual in maintenance of cellular homeostasis under stress conditions. Further, altered intracellular cations cause anomalous interactions with DNA and RNA and disrupt the structure and functions of nucleic acids [26]. The up regulated *GADD45B* gene indicated the DNA damage caused in *L. rohita* due to hyper salinity stress. In favor of this, there was enrichment of DE mRNAs in cell cycle, cellular senescence and apoptosis pathways, preventing the replication of cells with damaged DNA [31] under salinity stress.

As explained above, the cellular stress response towards high salt concentration is an energy demanding process. Hence, there was relocation of energy towards stress response specific functions [32]. There was enrichment of pathways such as oxidative phosphorylation, fructose and mannose metabolism, fatty acid biosynthesis, pentose phosphate pathway, glycolysis/gluconeogenesis and citrate cycle. Activation of these pathways under salinity stress, required for additional ATP production and reducing NAD(P)H + equivalents during stress [26]. Elongation of very long chain fatty acid 6 (*ELOVL6*) gene catalyzes the chain elongation and convert C12-16 saturated and monounsaturated fatty acids to C18 polyunsaturated fatty acids [33, 34] and [35] experiment of Chinese mitten crab proved that high dietary levels of polyunsaturated fatty acids significantly improved the salinity tolerance. Free fatty acid receptor 3 (*FFAR3*) plays an important role in lipid metabolism and regulation of plasma glucose [36]. Enolase 1 (*ENO1*) catalyzes 2-phosphoglycerate to phosphoenolpyruvic acid [37], a crucial step in glycolysis, whereas acetyl –CoA (*ACSS2*) is considered as direct energy precursor through citric acid cycle [38].

Enriched signal transduction pathways such as MAPK, FOXO and phosphatidylinositol signaling systems explained the role of signal transduction in ionic and osmotic regulation, and control of extracellular fluid volume. MAPK signaling is found to be an important pathway in salinity stress response [39]. However, signal transduction requires interaction between receptors and signaling molecules [40]. In *L. rohita* under hypersalinity stress, DE mRNAs were involved in cell adhesion molecules (CAMs), neuroactive ligand-receptor interaction, ECM-receptor interaction and cytokine-cytokine receptor interaction. Cells can also communicate through cell contact and gap junctions [41]. The analysis of KEGG enrichment indicated that focal adhesion and gap junction signaling pathways were involved in cellular communications. Focal adhesion, interacted cells to extracellular matrix (ECM) and gap junctions acted on cell to cell interaction [39]. These pathways concluded that in *L. rohita* under hyper salinity stress, sophisticated towards transmembrane transport and signal transduction.

Hyper salinity altered the immune response of *L. rohita*, there was differential expression of several innate immune response genes. Differentially expressed mRNAs involved in phagosome pathway were found to be up regulated including complement C3 like protein (*C3*), *SEC61A1*, *HLA-DRA*, *TAP2*, *CORO1A* and *HLA-A* genes. Phagocytosis is a pivotal cellular process in innate immune response and antigen presentation [42]. LRR and PYD-domains containing protein 3 (*NLRP3*) was found to be up regulated in salinity treated groups and observed enrichment of NOD-like signaling pathway. *NLRP3* is a pivotal gene that plays an important role in innate immune response, involved in release of cytokines and primary defense against microbes [43–45]. Cathepsin L (*CTSL*) [46] and NLR family member X1 (*NLRX1*) were observed to be involved in innate immune response [47]. *TRPM2* [48] and G-protein coupled receptor family C group 6 member A (*GPRC6A*) [49] were found to be important for the production of cytokines. From these findings, we hypothesized that the innate immune system acted in *L. rohita* under hyper salinity stress as a defensive mechanism against adverse environmental conditions [50].

## Conclusion

The present study gives insight into the regulatory network of lncRNA-miRNA-mRNA of *Labeo rohita* during the salinity stress. This study analysis revealed important lncRNAs and miRNAs associated with stress response in *Labeo rohita*, a non-model freshwater fish and it may serve as important markers for future studies in understanding the role of lncRNAs and miRNAs under hypersaline environmental conditions. TCONS_0071514-ipu-miR-205 pair found to regulated salinity adaptive response genes enriched in osmoregulation, signal transduction pathways and unfolded protein response. In addition, our study also revealed tni-miR-9 miRNA mediated the expression of genes involved in protein processing in endoplasmic reticulum, environmental information processing pathways and immune response. oni-miR-10,712 targeted inositol-3-phosphate synthase 1 A gene, key enzyme for osmolyte production. Genes involved in phagosome pathway are regulated by gmo-miR-459-39 miRNA, whereas dre-miR-152 regulated gene expression of energy metabolism. A few important lncRNA-miRNA pairs have been found in the study which helps the organism to cope up with hypersaline conditions. Further these study provides the basal expression data during osmotic imbalance which may be helpful to the researchers in the area working in the strain improvement and selection.

## Materials and methods

### Sample collection and salinity stress

The details of the salinity stress experimental design and sample collection has been published earlier [51]. In brief salinity stress experiment was conducted at Postgraduate Institute of Fisheries Education and Research (PGIFER), Kamdhenu University, Himmatnagar, Gujarat. Healthy *L. rohita* fingerlings (> 10 g) were acquired from a local freshwater farm and acclimatized to lab conditions in 150 L tanks (15 fingerlings/ tank) for seven days with continuous aeration at 27 ± 5 °C. Feeding was done three times a day at the rate of 5% body weight. Unused feed and fecal matter were siphoned out and 25% of water from the bottom of the tank was replaced daily. Later the fingerlings were randomly divided into control and salinity treatment groups. The experiment was done in triplicate. Control group was maintained at 0ppt throughout the experiment, while for the treatment group the salinity was gradually increased (1ppt/day) to specified salinity (2, 4, 6 and 8ppt) by adding a solution (55ppt) of Red Sea Coral Pro Salt (Red Sea, USA). The salinity was checked with a salinity refractometer RES-10ATC (ATC, USA). The fingerlings were maintained at a particular salinity for 6 days and on the 6^th^ day 3 fish were randomly sampled from the control and treatment groups. Then the salinity was raised to the next level and the process repeated until the last set of samples were collected at 8ppt on the 32^nd^ day from the start of the experiment. Similar survival of fingerlings was observed in control and treated groups. At each salinity gill tissue samples were dissected out aseptically and stored at -80 °C in RNAlater® until further use.

### RNA extraction, library preparation and Illumina sequencing

The total RNA was extracted from gill tissues using RNeasy Plus Mini Kit (Qiagen, Germany). The OD260/280 ratio and concentration were detected by the QIAxpert™ instrument (Qiagen, Germany) and Qubit 4 Fluorometer (Thermo Fisher Scientific, United States) respectively. The quantification of RIN value was assessed using Agilent 2100 Bioanalyzer system (Agilent technologies, California, United States). Depletion of rRNA was carried out with Low Input RiboMinus™ Eukaryote System v2 (Thermo Fisher, Massachusetts, United States) and library was prepared using TruSeq™ Total RNA Library Prep Kit (Illumina, California, United States). Transcriptome paired end (PE) sequencing was performed on Novaseq 6000 (2 × 150 bp read length) with a total yield of 335 Gbp.

### Mapping and identification of differentially expressed mRNAs (DE mRNAs)

FASTQ files were obtained from base call (bcl) files using argument (--bcl-conversion-only) in Illumina Dragen server. The quality of data was ensured using FASTQC tool. The data with Phred score (Q > 30) was mapped against the reference genome (GenBank assembly accession: GCA_004120215.1) with STAR alignment tool. The reads mapped to each gene in the genome were estimated using the FeatureCounts. Differentially expressed genes were obtained using the DESeq2 package with p-value < 0.05 and log2 (Fold Change) > 0.5 were applied as threshold to obtain significant genes. Enrichment and ontology of significant DEGs, GO and KEGG (Kyoto encyclopedia of genes and genomes database) (https://www.kegg.jp/kegg/kegg1.html) [52–54] was performed using KOBAS-i (v-3.0) with (Benjamini and Hochberg) as FDR correction method.

### Protein-protein interaction network analysis for identification of hub genes

Protein-protein interaction among DE mRNAs was established using STRING network analysis (https://string-db.org) with confidence interaction score of 0.4. Further, Cytoscape (3.9.1) was used to visualize the PPI network. CytoHubba (https://apps.cytoscape.org/apps/cytohubba), a plugin of cytoscape, was used to identify the hub genes based on maximal clique centrality (MCC) algorithm. MCC was reported to be the most effective algorithm in identification of hub genes with increased sensitivity and specificity [55]. In our study, genes with top 10 MCC scores were considered as hub genes.

### miRNA identification and differential expression of miRNA

Paired end fastq files were merged and converted to fasta format. Further, collapsed reads were generated from the merged fasta files. Collapsing identical reads is beneficial for miRNA identification because miRNA length (16–24 bp) is lesser than sequence length (150 bp) [56]. Identified putative mature miRNAs in *Labeo rohita* according to method followed by [57] with some modifications as discussed below. The sequences of the mature miRNAs were obtained from the miRBase database (https://www.mirbase.org) for all teleostei species. Mature miRNA sequences were used as query sequences for offline BLASTn against the generated collapsed reads with parameters i.e. E-value cut off 1E-1, percentage of identity ≥ 95. BLASTn output files were filtered based on miRNA sequences having minimum of 16 nt and maximum of 24 nt in length.

For differential expression of miRNA analysis, the read count matrix was computed from collapsed read files and analyzed with the EdgeR package from Bioconductor. P-value < 0.05 and log fold change (Log FC) > 0.5 or < -0.5 were used as threshold parameters to identify significantly differentially expressed miRNAs.

### miRNA target gene prediction and construction of miRNA-mRNA regulatory network

Potential targets for differentially expressed miRNAs were identified using miRanda (3.3a) tool, which works on local homology between mature miRNA-sequence and query target gene sequence. In this study, we used coding sequences of *Labeo rohita* as target sequence obtained from NCBI (GenBank assembly accession no: GCA_004120215.1_ASM412021v1). Differentially expressed mRNAs targeted by differentially expressed miRNAs were considered as miRNA-mRNA (gene) regulatory pairs for further analysis and miRNA-mRNA regulatory network was constructed by Cytoscape (v3.9.1).

### Prediction of lncRNA and differentially expressed lncRNAs (DE lncRNAs)

Aligned transcripts with STAR were used for de-novo assembly with Cufflinks version (v2.2.1) using mapping information. Combined assembly was obtained with Cuffmerge (v2.2.1) and merged assembly was processed through FEELnc (v.0.2.1) for identification of lncRNA using default parameters. Initially, transcripts less than 200 bp were filtered using FEELnc_filter_ which also identifies single-exon transcripts. Then, FEELnc_codpot_ was used to calculate coding potential of each transcript on the basis of length of ORF, sequence bias and length of transcripts used to distinguish lncRNA from mRNA. Then, FEELnc_classifier_ was used subsequently process identified lncRNA and classify them into genic, intergenic, containing, same strand, convergent, divergent, overlapping and nested. Finally, CPC2 (v0.1) which uses support vector machine for additional assessment method for identification of coding potential of transcripts. DESeq2 (v1.32.0) was used to identify the differentially expressed lncRNAs (DE lncRNAs) between control and treated samples and DE lncRNAS with p-value < 0.05 were considered as significant.

### Construction of ceRNA network

miRanda (3.3a) tool was used to predict lncRNA-miRNA pairs. Expression correlation between lncRNA-mRNA pairs was calculated using corr.test () function using R environment. The lncRNA-mRNA pairs with Pearson correlation coefficient (PCC) > 0.90 and p-value < 0.05 were selected. Among lncRNA-miRNA pairs and miRNA-mRNA pairs, if both lncRNA and mRNA are targeted by same miRNA with negative co-expression, were considered as lncRNA-miRNA-mRNA pairs and network was visualized using cytoscape (v3.9.1).

## Electronic supplementary material

Below is the link to the electronic supplementary material.

Supplementary Material 1

Supplementary Material 2

Supplementary Material 3

Supplementary Material 4

Supplementary Material 5

Supplementary Material 6

Supplementary Material 7

Supplementary Material 8

Supplementary Material 9

Supplementary Material 10

Supplementary Material 11

## Data Availability

RNA-seq data generated in this study have been submitted to NCBI Short Read Archive (SRA) i.e. G1C2- SRR23339905; G2C2- SRR23339891; G1T2- SRR23339895; G2T2- SRR23339901; G1C4- SRR23339904; G2C4- SRR23339890; G1T4- SRR23339894; G2T4- SRR23339900; G1C6- SRR23339897; G2C6- SRR23339903; G1T6- SRR23339893; G2T6- SRR23339899; G1C8- SRR23339896; G2C8- SRR23339902; G1T8- SRR23339892 and G2T8- SRR23339898.

## References

[1] Sarma K, Dey A, Kumar S, Chaudhary BK, Mohanty S, Kumar T (2020). Effect of salinity on growth, survival and biochemical alterations in the freshwater fish Labeo rohita (Hamilton 1822). Indian J Fish.

[2] Cui Q, Qiu L, Yang X, Shang S, Yang B, Chen M (2019). Transcriptome profiling of the low-salinity stress responses in the gills of the juvenile Pseudopleuronectes yokohamae. Comp Biochem Physiol Part D Genomics Proteomics.

[3] Jeppesen E, Beklio\uglu M, Özkan K, Akyürek Z. Salinization increase due to climate change will have substantial negative effects on inland waters: a call for multifaceted research at the local and global scale. Innov. 2020;1. 10.1016/j.xinn.2020.100030 PMC8454634 34557708

[4] Kulp SA, Strauss BH (2019). New elevation data triple estimates of global vulnerability to sea-level rise and coastal flooding. Nat Commun.

[5] Baliarsingh MM, Panigrahi JK, Patra AK (2018). Effect of salinity on growth and survivality of Labeorohita in captivity. Int J Sci Res.

[6] Shakir HA, Qazi JI, Chaudhry AS, Hussain A, Ali A (2013). Nutritional comparison of three fish species co-cultured in an earthen pond. Biol.

[7] Hwang PP, Lee TH (2007). New insights into fish ion regulation and mitochondrion-rich cells. Comp Biochem Physiol - A Mol Integr Physiol.

[8] Seo MY, Mekuchi M, Teranishi K, Kaneko T (2013). Expression of ion transporters in gill mitochondrion-rich cells in japanese eel acclimated to a wide range of environmental salinity. Comp Biochem Physiol Part A Mol \& Integr Physiol.

[9] Ou Y, Li J, Xie J, Ma Z, Chen Y, editors. others. Study on gill ultrastructure and respiratory area in early developmental stages of grey mullet (Mugil cephalus). South China Fish Sci. 2014;10:52–7.

[10] Wei XH, Ru SG, Xu L, Isoda H (2001). Structural and functional changes of euryhaline fish branchial chloride cell and hormonal regulation during seawater and freshwater adaptation. Mar Sci.

[11] Ou Y-J, Liu R-J, Li J-E, Cao S-H. Structural changes in mitochondrion-rich cells in the gills of artificial selected Trachinotus ovatus offspring under different salinities. 2013. 10.11813/j.issn.0254-5853.2013.4.0411 23913893

[12] Eddy SR (2001). Non–coding RNA genes and the modern RNA world. Nat Rev Genet.

[13] Qu K, Wang Z, Lin X, Zhang K, He X, Zhang H (2015). MicroRNAs: key regulators of endothelial progenitor cell functions. Clin Chim acta.

[14] Nelson BR, Makarewich CA, Anderson DM, Winders BR, Troupes CD, Wu F (2016). A peptide encoded by a transcript annotated as long noncoding RNA enhances SERCA activity in muscle. Sci (80-).

[15] Salmena L, Poliseno L, Tay Y, Kats L, Pandolfi PP (2011). A ceRNA hypothesis: the Rosetta Stone of a hidden RNA. language? Cell.

[16] Guo L-L, Song C-H, Wang P, Dai L-P, Zhang J-Y, Wang K-J (2015). Competing endogenous RNA networks and gastric cancer. World J Gastroenterol.

[17] Yan B, Zhao L-H, Guo J-T, Zhao J-L (2012). miR-429 regulation of osmotic stress transcription factor 1 (OSTF1) in tilapia during osmotic stress. Biochem Biophys Res Commun.

[18] Tian Y, Shang Y, Guo R, Chang Y, Jiang Y (2019). Salinity stress-induced differentially expressed miRNAs and target genes in sea cucumbers Apostichopus japonicus. Cell Stress Chaperones.

[19] Xia Y, Cheng J, Liu Y, Li C, Liu Y, Liu P (2022). Genome-wide integrated analysis reveals functions of lncRNA-miRNA-mRNA interactions in Atlantic salmon challenged by Aeromonas salmonicida. Genomics.

[20] Huo D, Sun L, Storey KB, Zhang L, Liu S, Sun J (2020). The regulation mechanism of lncRNAs and mRNAs in sea cucumbers under global climate changes: defense against thermal and hypoxic stresses. Sci Total Environ.

[21] Evans DH, Claiborne JB. Osmotic and ionic regulation in fishes. Osmotic and ionic regulation. CRC Press; 2008. 295–366.

[22] Watanabe T, Takei Y (2011). Molecular physiology and functional morphology of SO42–excretion by the kidney of seawater-adapted eels. J Exp Biol.

[23] Sacchi R, Li J, Villarreal F, Gardell AM, Kültz D (2013). Salinity-induced regulation of the myo-inositol biosynthesis pathway in tilapia gill epithelium. J Exp Biol.

[24] Van Anholt RD, Spanings FAT, Nixon O, Wendelaar Bonga SE, Koven WM (2012). The effects of arachidonic acid on the endocrine and osmoregulatory response of tilapia (Oreochromis mossambicus) acclimated to seawater and subjected to confinement stress. Fish Physiol Biochem.

[25] Bowlus RD, Somero GN (1979). Solute compatibility with enzyme function and structure: rationales for the selection of osmotic agents and end-products of anaerobic metabolism in marine invertebrates. J Exp Zool.

[26] Evans TG, Kültz D (2020). The cellular stress response in fish exposed to salinity fluctuations. J Exp Zool Part A Ecol Integr Physiol.

[27] Radons J (2016). The human HSP70 family of chaperones: where do we stand?. Cell Stress Chaperones.

[28] Motosugi R, Murata S (2019). Dynamic regulation of proteasome expression. Front Mol Biosci.

[29] Hosokawa N, You Z, Tremblay LO, Nagata K, Herscovics A (2007). Stimulation of ERAD of misfolded null Hong Kong $α$1-antitrypsin by golgi $α$1, 2-mannosidases. Biochem Biophys Res Commun.

[30] Ogen-Shtern N, Avezov E, Shenkman M, Benyair R, Lederkremer GZ (2016). Mannosidase IA is in quality control vesicles and participates in glycoprotein targeting to ERAD. J Mol Biol.

[31] Kammerer BD, Sardella BA, Kültz D (2009). Salinity stress results in rapid cell cycle changes of tilapia (Oreochromis mossambicus) gill epithelial cells. J Exp Zool Part A Ecol Genet Physiol.

[32] Sokolova IM, Frederich M, Bagwe R, Lannig G, Sukhotin AA (2012). Energy homeostasis as an integrative tool for assessing limits of environmental stress tolerance in aquatic invertebrates. Mar Environ Res.

[33] Matsuzaka T, Shimano H, Yahagi N, Yoshikawa T, Amemiya-Kudo M, Hasty AH (2002). Cloning and characterization of a mammalian fatty acyl-CoA elongase as a lipogenic enzyme regulated by SREBPs. J Lipid Res.

[34] Moon Y-A, Shah NA, Mohapatra S, Warrington JA, Horton JD (2001). Identification of a mammalian long chain fatty acyl elongase regulated by sterol regulatory element-binding proteins. J Biol Chem.

[35] Sui L, Wille M, Cheng Y, Sorgeloos P (2007). The effect of dietary n-3 HUFA levels and DHA/EPA ratios on growth, survival and osmotic stress tolerance of chinese mitten crab Eriocheir sinensis larvae. Aquaculture.

[36] Kimura I, Inoue D, Maeda T, Hara T, Ichimura A, Miyauchi S (2011). Short-chain fatty acids and ketones directly regulate sympathetic nervous system via G protein-coupled receptor 41 (GPR41). Proc Natl Acad Sci.

[37] Kang HJ, Jung S-K, Kim SJ, Chung SJ (2008). Structure of human $α$-enolase (hENO1), a multifunctional glycolytic enzyme. Acta Crystallogr Sect D Biol Crystallogr.

[38] Jankowska-Kulawy A, Klimaszewska-Łata J, Gul-Hinc S, Ronowska A, Szutowicz A (2022). Metabolic and Cellular compartments of Acetyl-CoA in the healthy and diseased brain. Int J Mol Sci.

[39] Cui W, Ma A, Huang Z, Wang X, Sun Z, Liu Z (2020). Transcriptomic analysis reveals putative osmoregulation mechanisms in the kidney of euryhaline turbot Scophthalmus maximus responded to hypo-saline seawater. J Oceanol Limnol.

[40] Bhattacharya S. Handbook of cell signaling. 2004.

[41] Kurt B, Kurtz L, Sequeira-Lopez ML, Gomez RA, Willecke K, Wagner C (2011). Reciprocal expression of connexin 40 and 45 during phenotypical changes in renin-secreting cells. Am J Physiol Physiol.

[42] Sokolovska A, Becker CE, Ip WKE, Rathinam VAK, Brudner M, Paquette N (2013). Activation of caspase-1 by the NLRP3 inflammasome regulates the NADPH oxidase NOX2 to control phagosome function. Nat Immunol.

[43] McCall SH, Sahraei M, Young AB, Worley CS, Duncan JA, Ting JP-Y (2008). Osteoblasts express NLRP3, a nucleotide-binding domain and leucine-rich repeat region containing receptor implicated in bacterially induced cell death. J Bone Miner Res.

[44] Qiao Y, Wang P, Qi J, Zhang L, Gao C (2012). TLR-induced NF-$κ$B activation regulates NLRP3 expression in murine macrophages. FEBS Lett.

[45] He Y, Hara H, Núñez G (2016). Mechanism and regulation of NLRP3 inflammasome activation. Trends Biochem Sci.

[46] Chiang Y-R, Wang L-C, Lin H-T, Lin JH-Y (2022). Bioactivity of orange-spotted grouper (Epinephelus coioides) cathepsin L: Proteolysis of bacteria and regulation of the innate immune response. Fish \& Shellfish Immunol.

[47] Ding X, Liang Y, Hou Q, Li S, Peng W, Li R (2023). Molecular cloning and characterization of NLRX1 from orange-spotted grouper (Epinephelus coioides) as a mediator of ROS production through its LRR domains. Aquaculture.

[48] Wehrhahn J, Kraft R, Harteneck C, Hauschildt S (2010). Transient receptor potential melastatin 2 is required for lipopolysaccharide-induced cytokine production in human monocytes. J Immunol.

[49] Pi M, Nishimoto SK, Quarles LD (2017). GPRC6A: Jack of all metabolism (or master of none). Mol Metab.

[50] Narnaware BYK, Kelly SP, Woo NYS (2000). Effect of salinity and ration size on macrophage phagocytosis in juvenile black sea bream (Mylio macrocephalus). J Appl Ichthyol.

[51] Harshini V, Shukla N, Raval I, Kumar S, Shrivastava V, Patel AK (2022). Kidney transcriptome response to salinity adaptation in Labeo rohita. Front Physiol.

[52] Kanehisa M, Goto S (2000). KEGG: kyoto encyclopedia of genes and genomes. Nucleic Acids Res.

[53] Kanehisa M, Sato Y, Kawashima M, Furumichi M, Tanabe M (2016). KEGG as a reference resource for gene and protein annotation. Nucleic Acids Res.

[54] Moriya Y, Itoh M, Okuda S, Yoshizawa AC, Kanehisa M (2007). KAAS: an automatic genome annotation and pathway reconstruction server. Nucleic Acids Res.

[55] Chin CH, Chen SH, Wu HH, Ho CW, Ko MT, Lin CY (2014). cytoHubba: identifying hub objects and sub-networks from complex interactome. BMC Syst Biol.

[56] Baras AS, Mitchell CJ, Myers JR, Gupta S, Weng LC, Ashton JM (2015). MiRge - A multiplexed method of processing small RNA-seq data to determine MicroRNA entropy. PLoS ONE.

[57] Mastakani FD, Pagheh G, Monfared SR, Shams-Bakhsh M (2018). Identification and expression analysis of a microRNA cluster derived from pre-ribosomal RNA in Papaver somniferum L. and Papaver bracteatum L. PLoS ONE.

