# Supplementary material for: Interplay of gene expression and regulators under salinity stress in gill of Labeo rohita
Source: BMC Genomics. 2023 Jun 19;24:336. doi: 10.1186/s12864-023-09426-x (PMC10278310; doi:10.1186/s12864-023-09426-x)
Supplement: Supplementary file 1 — Supplementary Material 1 [file 12864_2023_9426_MOESM1_ESM.docx]

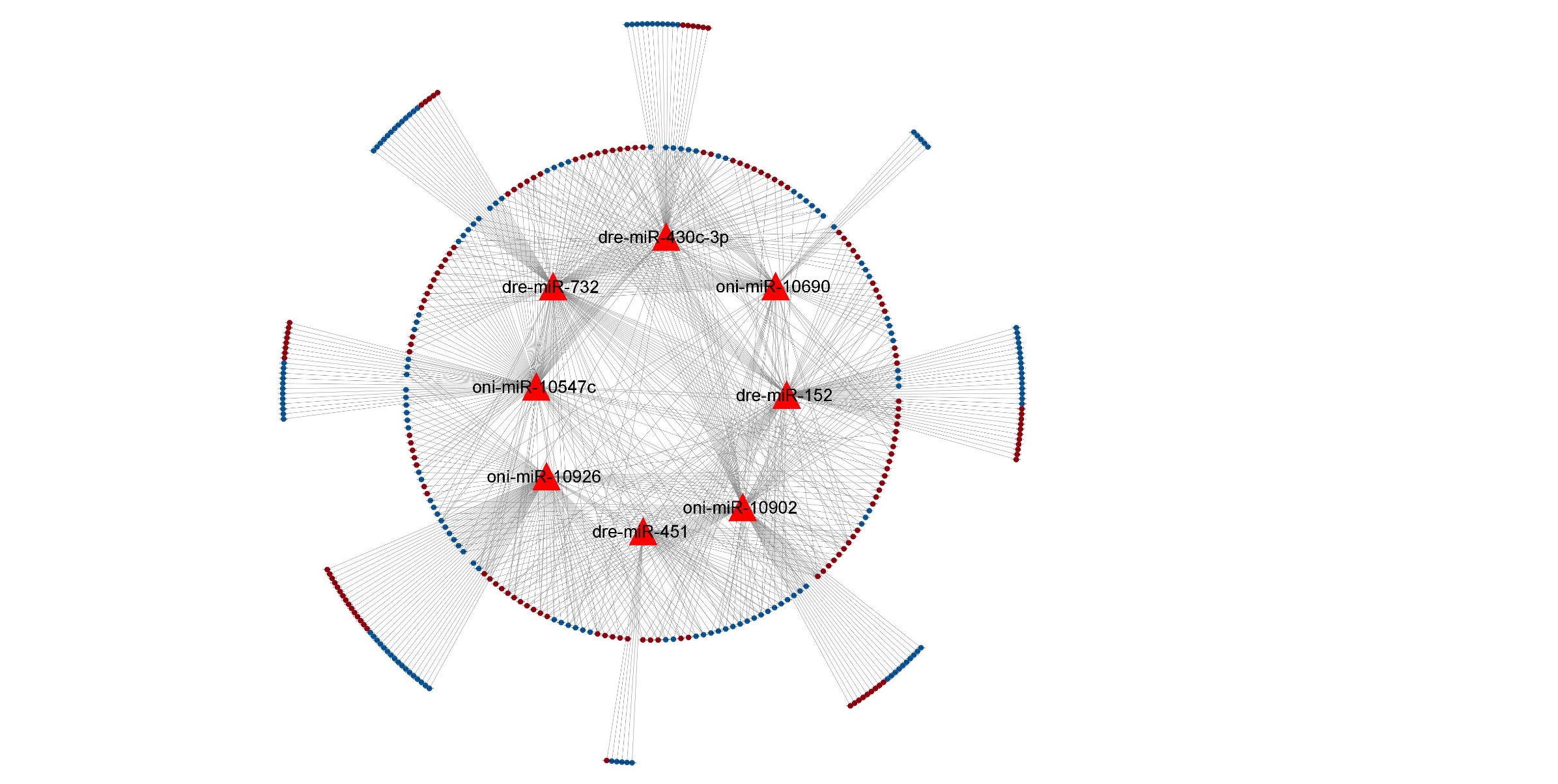

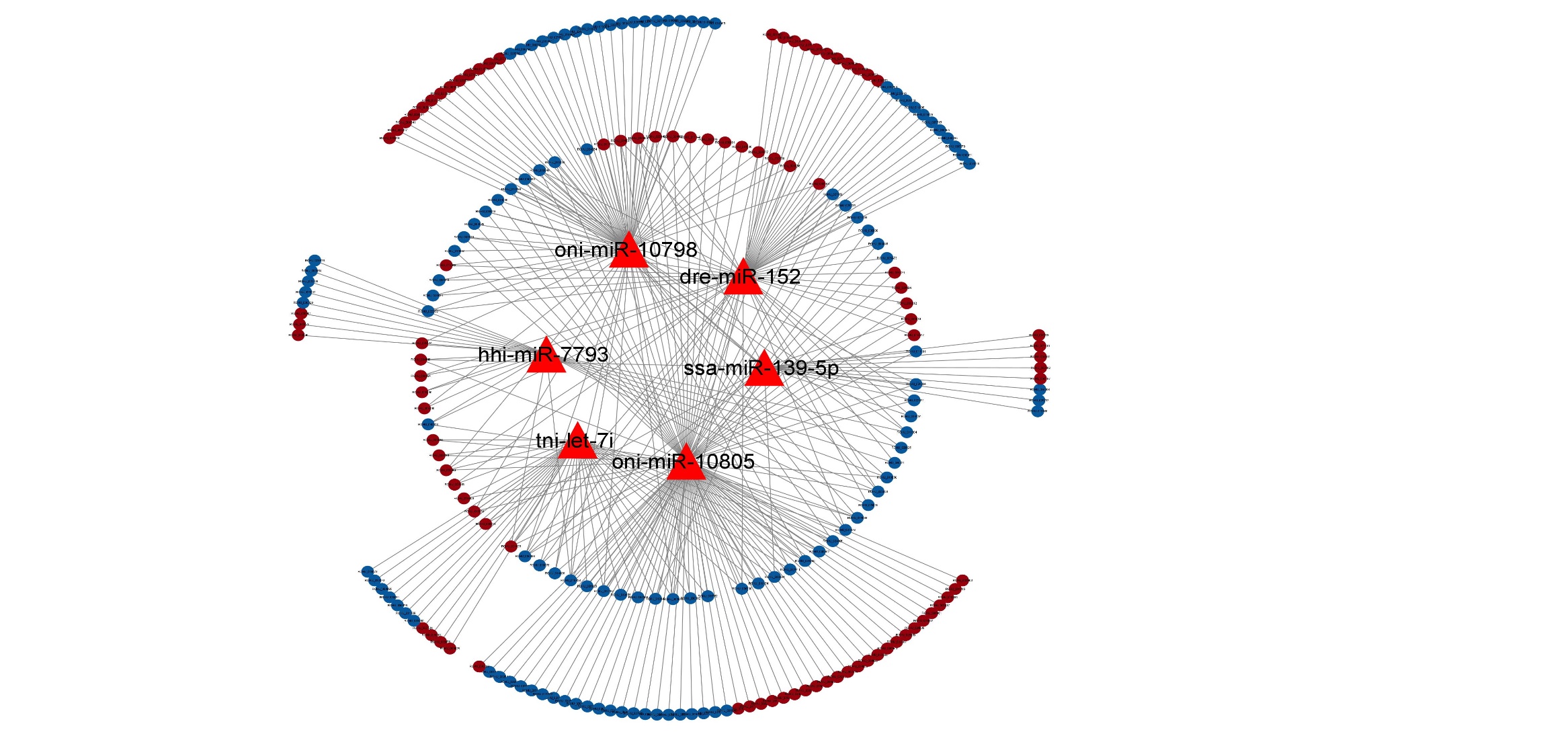


**SF1b**

**SF1a**

Fig.SF1. miRNA-mRNs regulatory network of (SF1a) 2ppt and (SF1b) 4ppt salinity treated groups of *Labeo rohita* gill transcriptome. The bright red colour triangle represents DE miRNAs, dark red colour elliptical shape represents up regulated DE mRNAs, while dark blue colour represents down regulated DE mRNAs. Each node represents individual miRNA/mRNA The generated network for 2ppt salinity treated group has 217 nodes and 337 edges, whereas 4ppt treatment group has 347 nodes and 688 edges.


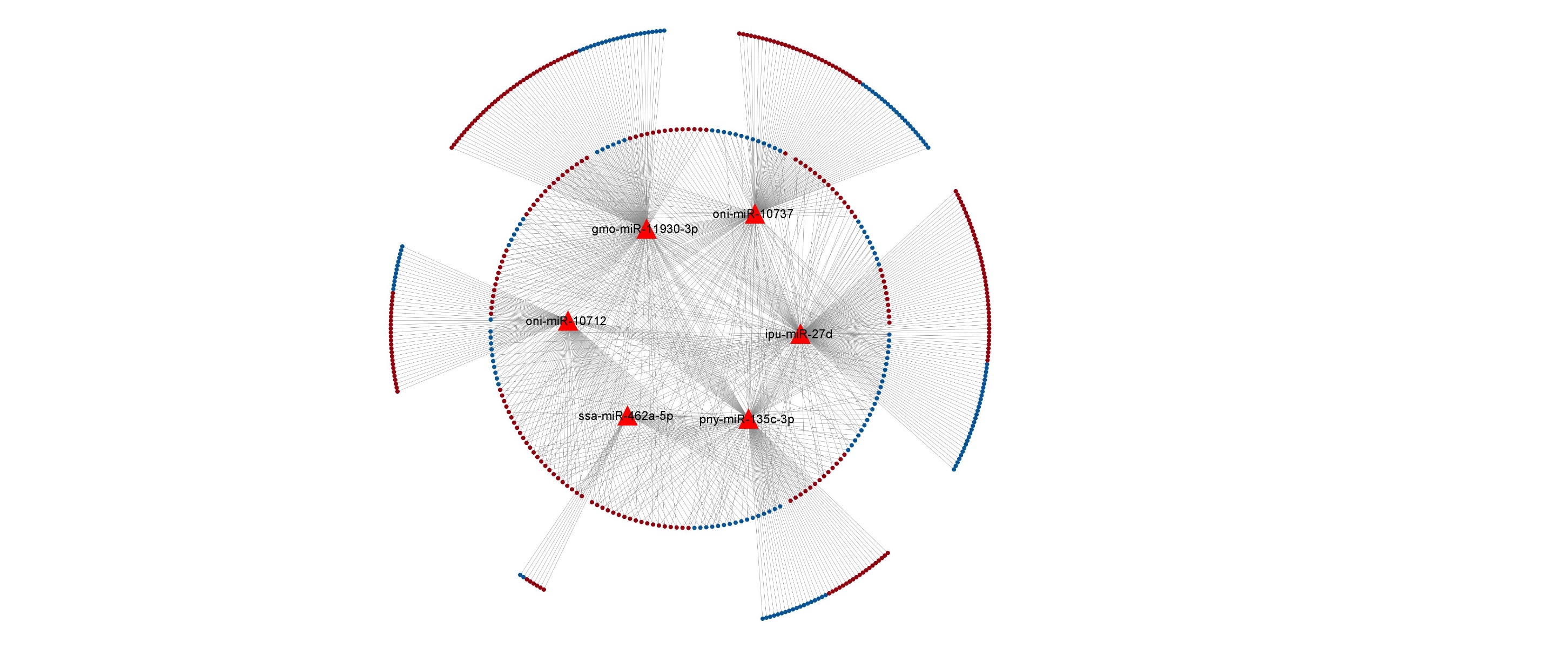

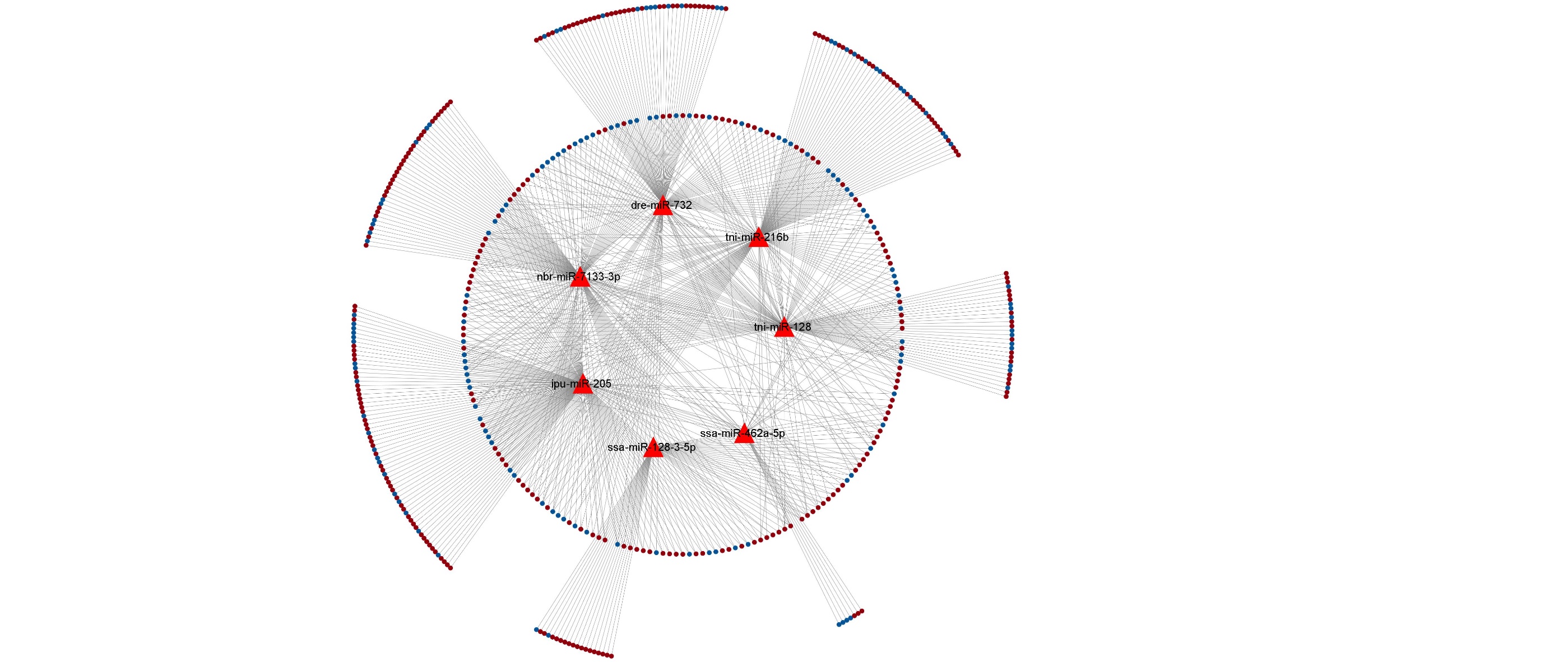


**SF2b**

**SF2a**

Fig.SF2. miRNA-mRNs regulatory network of (SF2a) 6ppt and (SF2b) 8ppt salinity treated groups of *Labeo rohita* gill transcriptome. The bright red colour triangle represents DE miRNAs, dark red colour elliptical shape represents up regulated DE mRNAs, while dark blue colour represents down regulated DE mRNAs. Each node represents individual miRNA/mRNA The generated network for 6ppt salinity treated group has 457 nodes and 764 edges, whereas 8ppt treatment group has 490 nodes and 781 edges.
